# Haplotype frequency estimation error analysis in the presence of missing genotype data

**DOI:** 10.1186/1471-2105-5-188

**Published:** 2004-12-01

**Authors:** Enda D Kelly, Fabian Sievers, Ross McManus

**Affiliations:** 1 Hitachi Dublin Lab., Hitachi Europe Ltd., O'Reilly Institute, Trinity College, Dublin 2, Ireland; 2 Dept. of Clinical Medicine, Trinity College Dublin and Dublin Molecular Medicine Centre at St. James's Hospital, Dublin, Ireland

## Abstract

**Background:**

Increasingly researchers are turning to the use of haplotype analysis as a tool in population studies, the investigation of linkage disequilibrium, and candidate gene analysis. When the phase of the data is unknown, computational methods, in particular those employing the Expectation-Maximisation (EM) algorithm, are frequently used for estimating the phase and frequency of the underlying haplotypes. These methods have proved very successful, predicting the phase-known frequencies from data for which the phase is unknown with a high degree of accuracy. Recently there has been much speculation as to the effect of unknown, or missing allelic data – a common phenomenon even with modern automated DNA analysis techniques – on the performance of EM-based methods. To this end an EM-based program, modified to accommodate missing data, has been developed, incorporating non-parametric bootstrapping for the calculation of accurate confidence intervals.

**Results:**

Here we present the results of the analyses of various data sets in which randomly selected known alleles have been relabelled as missing. Remarkably, we find that the absence of up to 30% of the data in both biallelic and multiallelic data sets with moderate to strong levels of linkage disequilibrium can be tolerated. Additionally, the frequencies of haplotypes which predominate in the complete data analysis remain essentially the same after the addition of the random noise caused by missing data.

**Conclusions:**

These findings have important implications for the area of data gathering. It may be concluded that small levels of drop out in the data do not affect the overall accuracy of haplotype analysis perceptibly, and that, given recent findings on the effect of inaccurate data, ambiguous data points are best treated as unknown.

## Background

Haplotype analysis has become a valuable tool for researchers in population genetics. In particular, the value attached to the prediction of the constituent haplotypes of a given sample and their frequency of occurrence is such that a variety of methods have been developed for this purpose. Many of these methods, however, depend on knowledge of the phase of the data supplied. In general, genotypic data from polymorphic loci are ascertained phase-unknown. Various methods for determining the gametic phase exist. With sufficient data from the genotyping of family members, definitive haplotypes may be inferred. However, in particular for late-onset disorders, these data may be difficult or even impossible to obtain. At the laboratory level, techniques such as chromosomal isolation or long-range PCR [1] may be utilised in the prediction of haplotypes, but they suffer the dual drawbacks of being both technologically demanding and in many cases prohibitively expensive in practice. Thus researchers have moved towards computational solutions to this problem. Prominent among the techniques employed for the estimation of the true haplotype frequencies of a phase-unknown sample are those based on the Expectation-Maximisation (EM) algorithm. Hill [2] originally proposed the use of the EM algorithm in genetics, and three years later the term was first coined by Dempster et al. [3] and the method put on a more formal footing. A number of EM-based methods for haplotype frequency estimation (HFE) have been produced [4,5]. Excoffier and Slatkin [6] provide a thorough outline of the implementation of the EM algorithm as applied to the problem of HFE.

Reliable computational techniques for the estimation of haplotype frequencies have been around for some time, and extensive studies of the accuracy of the EM-based methods have been carried out [7,8], but until recently there has been little investigation of the effect of missing data on these techniques. This is surprising considering that, even with modern automated DNA analysis methods, the problem of missing data is not uncommon, whether due to the failure of amplification or insufficient DNA. Zhao et al. [9] have developed the GENECOUNTING software specifically to take into account missing data in a sample, but have not produced any validation of the method. The HAPLO [5] program is also capable of analysing multiallelic data with missing alleles, using jackknife techniques for error analysis. The SNPHAP [10] algorithm can handle large numbers of loci and unknown alleles, but is restricted to the analysis of biallelic loci. In order to carry out an investigation of the effect of missing data on HFE, a program, based on the algorithm outlined in [6], has been developed which can accommodate multiallelic loci and a significant percentage of unknown alleles. The necessary alterations to the existing implementation of the EM algorithm are outlined in the Methods section. Following this, biallelic and multiallelic data sets were analysed with varying quantities of unknown alleles randomly substituted. The analysis is similar to previous work by Kirk and Cardon [11], which described the effect of genotyping error on HFE. Here we investigate the effect of missing data on the sizes of the confidence intervals (CIs) about the haplotype frequency point estimates (or simply "point estimates"). Surprisingly, the loss of as much as 30% of the allelic data did not have a significantly detrimental effect on the quality of the results. The frequencies of haplotypes which predominate in the complete data analysis remain essentially the same after randomly selected data have been relabelled as missing. The error estimates associated with the predicted frequencies, which are generated via a bootstrap method, are also quite stable, but increase as the proportion of missing data increases.

## Results

### Source of data

Two sources of data were used for the principal part of this study. The first is real single nucleotide polymorphism (SNP) data; the second is multiallelic data generated via population generation software. Three additional sets of data containing 10%, 20% and 30% missing alleles respectively were generated from each of the two original sets. The process of generation is described in the Methods section. HFE was carried out on the eight data sets listed above. In each case 1,000 bootstrap iterations were performed for each HFE analysis and the 95% CIs about the point results were selected. For the sake of clarity the results from analyses of the 20% unknown alleles data sets have been omitted from the displayed graphs. Further tests were performed to investigate the effect of sample size upon the quality of the results. To this end two sets of progressively smaller data sets, with and without missing alleles, were generated from the SNP and multiallelic data sets, and HFE was carried out. The method of selecting these data is outlined in the Methods section.

An additional data set, unrelated to those previously described, consisting of data from five SNP loci was generated for the purposes of performing tests on data with weak LD between the loci. A further data set with 10% missing alleles was generated from these additional data.

### Seven loci biallelic data sets

**Figure 1:**
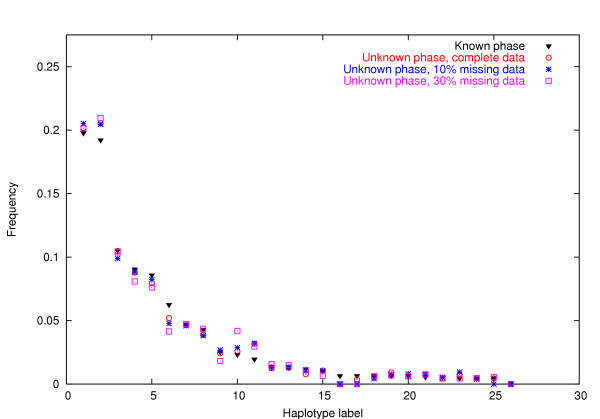
**Point estimates for seven loci SNP data**. Point estimate haplotype frequencies for seven loci SNP data set for phase-known and phase-unknown data with 0%, 10%, and 30% missing data.

**Figure 2:**
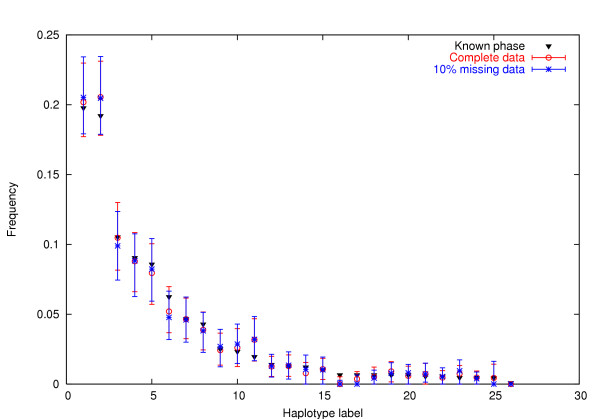
**Seven loci SNP data with 10% missing alleles**. 95% CIs for haplotype frequencies estimated from seven loci SNP phase-unknown data with 0% and 10% missing data. Frequencies derived from phase-known data also shown.

**Figure 3:**
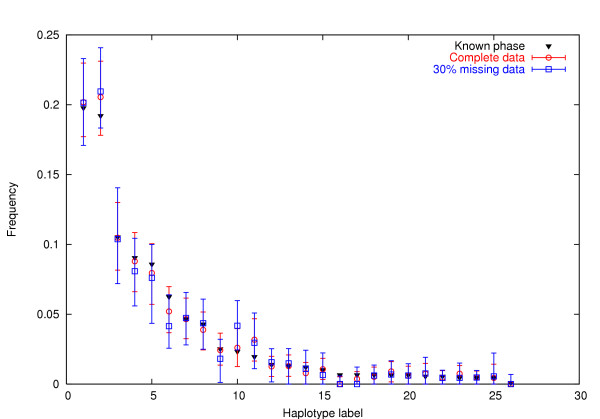
**Seven loci SNP data with 30% missing alleles**. 95% CIs for haplotype frequencies estimated from seven loci SNP phase-unknown data with 0% and 30% missing data. Frequencies derived from phase-known data also shown.

Figure 1 displays point estimate results from the analyses of the seven loci SNP data sets with 536 sample points. Figure 1 is a comparison of the frequencies of the 26 haplotypes present in the phase-known data, and their predicted frequencies when the phase is assumed unknown and data are missing. The percentage of missing alleles varies from zero (labelled "complete data") to 30. The haplotypes derived from the phase-known data were labelled from 1 to 26 in non-increasing order of the magnitude of their frequency, hence the "haplotype label" of the x-axis. For a quantitative measure of the discrepancies in the frequencies between the phase-known and phase-unknown frequency predictions we use the measure *D *(*h*, 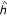) [6,11] given by


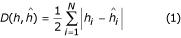


where *h*_*i *_and 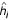 are the haplotype frequencies derived from the phase-known and phase-unknown data respectively, and *N *is the number of possible haplotypes in the sample. As these data are from seven biallelic loci, *N *= 2^7 ^= 128 in this case. The results are displayed in Table 1. Also recorded in Table 1 is the percentage increase in *D *(*h*, 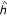) as the percentage of unknown alleles in the sample increases. In each case it is the percentage increase relative to the complete data value that is measured. Three haplotypes absent from the phase-known data set appear in the results of the HFE analysis of the complete data. Their frequencies are 2.3 × 10^-3^, 1.4 × 10^-3^, and 1.1 × 10^-3^. Of the haplotypes present in the phase-known data, only one haplotype appears with a frequency less than these, the given frequency being 9.3 × 10^-4^. Figure 1 and Table 1 offer complementary illustrations of the effect of missing data. Table 1 provides a good overall picture of how the accuracy of the HFE method deteriorates with inferior data quality. The effect is most marked in the initial jump from complete to 10% missing data, where a 35% increase in *D *(*h*, 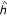) is recorded. The subsequent percentage increases going from 10% to 20% and 20% to 30% unknown alleles are 22% and 16%, respectively, of the value of *D *(*h*, 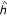) for the complete data. Figure 1 allows us to view specifically where this deterioration is most evident, in the mid-range frequency haplotypes. Figures 2 and 3 display the effect of increasing quantities of missing data on the 95% CIs of the haplotype frequencies estimated from the phase-unknown data. In an attempt to quantify this effect, the spread of the CIs for each haplotype (the difference between the two bootstrap haplotype frequencies which give the limits of the 95% CI) was summed for each data set. The sum for each data set containing missing data was compared with the sum for the complete data set (no missing data). The ratio of the two values (the ratio of the extent of the CIs or RCI) for each comparison is displayed in Table 2. Here we see a superlinear increase in the RCI with increasing proportions of missing data. Despite this, we note from Figures 2 and 3 that, even for the 30% missing data case, the CIs for the complete data are not entirely contained within the CIs for the data with unknown alleles for many of the haplotypes.

### Multiallelic data sets

**Figure 4:**
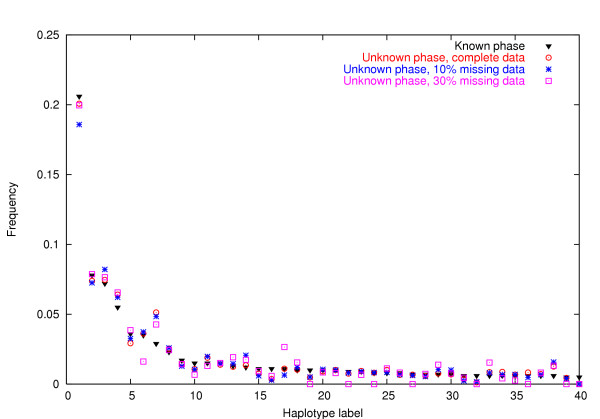
**Point estimates for multiallelic data**. Point estimate haplotype frequencies for multiallelic data set for phase-known and phase-unknown data with 0%, 10%, and 30% missing data.

**Figure 5:**
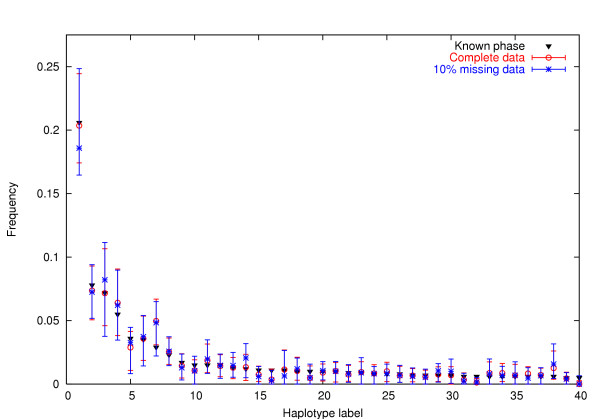
**Multiallelic data with 10% missing alleles**. 95% CIs for haplotype frequencies estimated from multiallelic phase-unknown data with 0% and 10% missing data. Frequencies derived from phase-known data also shown.

**Figure 6:**
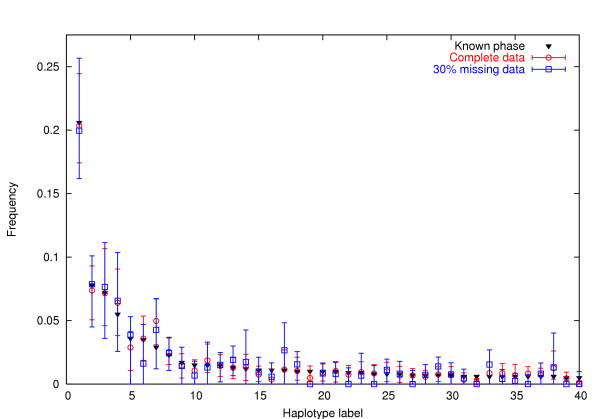
**Multiallelic data with 30% missing alleles**. 95% CIs for haplotype frequencies estimated from multiallelic phase-unknown data with 0% and 30% missing data. Frequencies derived from phase-known data also shown.

Similar computations to those carried out for the SNP data sets were carried out for the four multiallelic data sets. Figure 4 is a comparison of the frequencies of the most prominent haplotypes in the phase-known data, and their predicted frequencies when the phase is assumed unknown and data are missing. As with the seven loci SNP data sets, the percentage of missing data varies from zero to 30. The haplotypes are labelled as before. However, as 118 distinct haplotypes appear in the phase-known data, only the frequencies for the 40 most common are illustrated in Figures 4 to 6 for reasons of clarity. No haplotype with a frequency greater than 0.005, as given by the phase-known data, was excluded from the graphs by this trimming. As with the biallelic data, the discrepancy between the phase-known and phase-unknown frequency predictions, *D *(*h*, 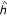), was measured. As the allele counts at each of the seven loci are 8, 2, 2, 9, 2, 5, and 2 respectively, the sum in Equation 1 is over the *N *= 5760 possible haplotypes in the sample. The results are displayed in Table 3. As in Table 1, the percentage increase in *D *(*h*, 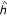) as the percentage of unknown alleles in the sample increases is also recorded.

129 distinct haplotypes were estimated to have a frequency of greater than 10^-6 ^as a result of the HFE analysis. 29 of these do not appear in the phase-known data, with the most common of these having a frequency of 2.187 × 10^-3^. 68 haplotypes in the phase-known data display a frequency greater than this. As with the SNP case, Figure 4 and Table 3 together provide a good overall picture of the effect of missing data on the accuracy of the HFE method. Table 3 displays similar percentage increases in *D *(*h*, 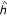) with the 10% and 20% missing data cases to those of Table 1 (42% and 18% respectively), though there the similarity ends, as the jump in *D *(*h*, 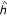) going from 20% to 30% unknown alleles comes to 40% of the value of *D *(*h*, 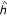) for the complete data. In Figure 4 we see how the phase-unknown frequency predictions match well the observed phase-known frequencies for the more prominent haplotypes, but less well for the less common haplotypes, particularly for the 30% missing data case.

Similarly to the SNP case, Figures 5 and 6 display the effect of increasing quantities of missing data on the 95% CIs of the haplotype frequencies estimated from the phase-unknown data. As before, measurement of this effect was made by observing the relative increase in the sizes of the CIs. The results are displayed in Table 4. In contrast to the SNP case, we see a linear increase in the RCI with increasing proportions of missing data. This contrast is further marked by Figures 5 and 6 where we note that the CIs for the complete data are, in the case of most haplotypes, entirely contained within the CIs for the data with unknown alleles.

### Sample sizes

Investigations were made into the effect of the sample size on the performance of the HFE method when 10% of the data was missing. Three further data sets of sizes 300, 100 and 50 individuals were generated by random selection from the original seven loci SNP and multiallelic sets. From these data, six additional sets with 10% missing alleles were created. HFE was performed upon these additional data, and the *D *(*h*, 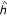) results for each were displayed in Table 5. In each case the phase-known haplotype frequencies used in the computation of *D *(*h*, 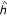) were those derived from the respective smaller samples (e.g. the accuracy of the HFE method as applied to the SNP sample with 300 individuals was calculated relative to the haplotype frequencies observed in the phase-known sample with 300 individuals, and not those observed in the original data). As may be expected, in all cases we see an increase in *D *(*h*, 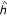) as we move from the complete data to the data sets with missing alleles. *D *(*h*, 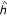) also is seen to increase as the sample size decreases. However, what is of note is the pattern involved. For the seven loci SNP case, the percentage increase in *D *(*h*, 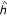) from complete to missing data itself increases monotonically as the sample size is reduced. A similar pattern is not observed in the multiallelic data.

### Performance at low LD levels

Fallin and Schork [7] illustrate how the performance of the EM-based HFE method diminishes with falling LD strength. Here we investigated how the accuracy of our implementation behaves on a data set exhibiting weak LD when 10% of the alleles are missing. A population of 500 individuals with data at five SNP loci was generated specifically for this part of the study. Lewontin's *D*' [12] was found to range between 0.117 and 0.014 for all adjacent loci. Table 6 displays *D *(*h*, 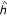) readings for this particular case. Here we see a large percentage increase of 60% in *D *(*h*, 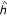) as we move from the complete data to 10% missing data.

## Discussion

The results displayed here show the impact of the addition of increasing quantities of missing alleles on the quality of haplotype frequency estimates. Studying Figure 1 in tandem with Table 1, and Figure 4 in tandem with Table 3, we see a loss of accuracy of the HFE method as the quality of the data degrades. This is particularly true for the multiallelic data set with 30% missing alleles. Here the loss of accuracy is most apparent with the rarer haplotypes as may be seen in Figure 4, whereas for the seven loci SNP case, Figure 1 illustrates that the low frequency haplotypes are dealt with remarkably well, even at high missing data proportions. For both sets of data the ability of the method to predict the frequencies of the most prominent haplotypes in the samples holds up well as the percentage of unknown alleles increases. Figures 2 and 3 and Figures 5 and 6 display a similar behaviour in the bootstrap generated CIs. To summarise, there are two significant aspects of the analysis of genotypic data containing incompletely typed individuals evident here. Firstly, that the HFE algorithm, given phase-unknown data with moderate to high levels of LD, predicts the frequencies of the underlying haplotypes with a high degree of accuracy, as is evident from the point estimate graphs, Figures 1 and 4. Tables 1 and 3 quantify how the quality of the frequency predictions behave with increasing percentages of missing data. For the multiallelic case where 30% of the alleles are unknown, Table 3 shows that the discrepancy between the phase-known and phase-unknown predicted frequencies has doubled when compared with the complete data case, though from the study of Figure 4 the bulk of this discrepancy would appear to originate from the lower frequency haplotypes. The second aspect is the extent of the 95% CIs. We see a steady increase in the spread of the CIs with the addition of missing alleles, reflecting the growing uncertainty in the data. However, the most prominent haplotypes in both the SNP and multiallelic data sets maintain their distinctiveness, even at the 30% unknown alleles level. These data show that, in particular for the SNP data set, the effect of relabelling significant proportions of the data as unknown on the performance of the HFE algorithm is minor.

Although study of the illustrated graphs suggests that the impact of missing data is more pronounced with the more complex multiallelic data sets, Tables 2 and 4 demonstrate that the relative increase in the size of the CIs is similar across the biallelic and multiallelic data sets, and is almost identical for the 30% missing data sets. There appears to be a discrepancy between the two measures, namely *D *(*h*, 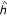) and the RCI, used here to quantify the degradation in the quality of the results with increasing percentages of unknown alleles. Tables 1 and 3 imply that the HFE method works significantly better for biallelic data than for multiallelic data, whereas this phenomenon is much less evident in Tables 2 and 4. This may be explained by the fact that *D *(*h*, 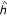) is an *absolute *measure of the performance of the algorithm, as the phase-known data are available for each data set and thus the exact sample haplotype frequencies are known. This discrepancy is to be expected; *D *(*h*, 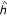) is a sum over all possible haplotypes and there exist only 128 (2^7^) possible haplotypes for the seven loci SNP data, whereas the multiallelic data, as noted in the Results section, have 5760 possible haplotypes. Also, it is not surprising that haplotype frequencies estimated from the multiallelic data set are found to be less accurate than those estimated from SNPs, given the more complex nature of the data. The RCI is a relative measure, and illustrates not so much the accuracy of the algorithm, rather the effect of additional missing data. The results displayed in Tables 2 and 4 show that the algorithm handles the increase in the proportion of unknown alleles equally well for both SNPs and multiallelic data, although it should be pointed out that the RCI measure gives no indication of the accuracy of the point estimates, and should generally be considered in tandem with a measure such as *D *(*h*, 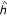). Interestingly, the results for the multiallelic data set were achieved despite departure from Hardy-Weinberg equilibrium (HWE) at two of the seven loci (see Methods section). Although this technique relies on the assumption of HWE, Niu et al. [13] have demonstrated it to be reliable and robust even when the HWE assumption has been violated. Fallin and Schork [7] have shown that HWE violation which results in an excess of heterozygosity leads to an increase in HFE error, though their results are based on a five-locus system, and the observed error increase when two of the five loci were found to be in disequilibrium was minimal. As we are dealing here with a seven-locus system, the effect on the error was likely to have been even less apparent.

The investigation into the effect of smaller sample sizes has produced some surprising results. Comparing Table 1 with Table 5, we see that the relative increase in *D *(*h*, 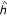) observed when 10% of the seven loci SNP data is relabelled as missing does not change substantially as the size of the sample reduces. For the full sample of 536 individuals, the percentage jump in *D *(*h*, 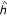) moving from the complete data to 10% missing data is approximately 35%. For the sample of size 300, this increase is 37%. Likewise for the samples of size 100 and 50, the increases are 41% and 47% respectively. However, for the multiallelic data, we see a contrasting trend. The percentage jump in *D *(*h*, 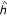) decreases rather than increases with increasing missing data proportions. Inspection of Tables 3 and 5 shows us that the percentage increase in *D *(*h*, 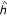) when moving from the complete data to 10% missing data for the full sample of 500 individuals is approximately 42%, whereas for the sample of size 300 this drops to 32%. The recorded increase for the sample of size 100, 5%, is even more striking. (The sample of size 50 is not considered here, as the matching observed between the phase-known and phase-unknown frequencies was of poor quality (figure not shown), and any conclusions drawn from analysis of this case would be highly suspect). Thus no definitive conclusions may be made as to the effect of missing data as the sample size is reduced, other that to say that the matching between the phase-known and phase-unknown frequencies deteriorates with falling sample size, as would be expected.

Table 6 underlines the relationship between strong LD and superior performance of the EM method [7]. For the weak LD data set, we see that *D *(*h*, 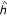) for the complete data is comparable to that of the seven loci SNP data with 30% missing alleles. It should also be borne in mind that, as the weak LD data set features only five SNP loci, the sum for *D *(*h*, 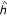) is over a mere 32 possible haplotypes, as compared to 128 for the seven loci SNP data, emphasising the fall-off in accuracy. Also of note is the similarity in the sample sizes -500 in the weak LD case, and 536 in the moderate to strong LD case. Moving to the 10% missing allele case, we witness a further 60% drop in accuracy, a considerably greater percentage that was observed for the medium to high LD data sets, a result which again calls into question the reliability of the method in the presence of weak LD.

## Conclusions

Here we show that the EM method, with the modifications to the implementation for complete data detailed here, can generate accurate estimates of haplotype frequencies even when large amounts of data are missing, in this case up to 30%. Moreover, using this method, the degree of accuracy can easily be estimated using conventional bootstrapping approaches. This is of considerable importance in the design of experiments, as it is therefore obvious that small levels of drop out in the data for whatever reason do not affect the overall accuracy of the approach perceptibly. Furthermore, considering the strongly deleterious effects of even small amounts of inaccurate data [11], this analysis shows that large amounts of missing data are much less detrimental to the overall quality of the results than incorrectly typed sites. Thus from a practical standpoint it is clearly preferable that if any doubt exists as to a genotype's identity, it should be excluded rather than included using a "best guess".

## Methods

### Seven loci biallelic data

The data used in this part of the study are derived from a genetic investigation of cystic fibrosis sufferers [14]. The haplotypes used here are actual haplotypes composed of a subset of the markers typed in the vicinity of the CFTR gene locus. The haplotypes comprise seven biallelic loci. From these haplotypes 536 phase-known genotypes were constructed via random resampling. Thus the data set comprised of 536 individuals each with seven SNP loci. In common with Kirk and Cardon [11] a linkage disequilibrium (LD) analysis was carried out on the data. For adjacent loci, *D*' was found to be ≥ 0.9 for all intervals but the third and fifth, where *D*' ≤ 0.25. As HWE is assumed for HFE, each locus was tested and found to be in HWE.

### Multiallelic data

An initial population of fifty individuals with data from seven loci spaced 1 cM apart was generated *in silico*. The number of distinct alleles at each locus ranged from two to nine. A trait marker was introduced between the 3^rd ^and 4^th ^loci for 10 of the 50 founders. The population was evolved for thirty generations as an isolated group with random mating. The birth rate per couple was binomially distributed, with a range of zero to ten offspring and a mean of 2.5. 500 individuals bearing the trait were randomly selected from the final generation for analysis. As with the SNP data, the level of LD across the interval was measured. *D*' was found to lie between 0.5 and 0.8 for all adjacent loci except between the second and third loci where *D*' = 1.0 and the fifth and sixth where *D*' = 0.24. A test for HWE [15] was performed, and it was found that the fourth and fifth loci were not in HWE (P-values < 0.001 and 0.003 respectively). In both cases an excess of heterozygosity was evident (observed heterozygosities of 0.848 and 0.232, respectively compared with expected heterozygosities of 0.729 and 0.205, respectively).

### Smaller sample sizes

The data sets of reduced size used in this analysis were generated from the original seven loci SNP and multiallelic data sets via a random sampling process. The process was identical for both. Initially 300 individuals were chosen from the original data. Following this, 100 individuals were chosen from the newly created set of size 300. Finally 50 individuals were chosen from the set of size 100. In each case the selection process was random and done without replacement. From each of these six smaller data sets, six additional sets of data with 10% missing alleles were generated by the process outlined below.

### Low LD data

A population of 500 individuals with data from five SNP loci was generated *in silico *specifically for the testing of the performance of the HFE algorithm in low LD circumstances. *D*' was found to range between 0.117 and 0.014 for all adjacent loci. The data were also tested for HWE. The first locus was found to be marginally not in HWE (P = 0.0465), with excess homozygosity in evidence. All other loci were found to be in HWE.

### Phase-unknown data

The HFE algorithm assumes that the input data are phase-unknown, and thus no alteration was necessary to the sample data sets which were phase-known before input. Comparison tests on the phase-known data, and phase-unknown data generated from the phase-known data via a process of phase-randomisation have confirmed that no bias is introduced by the use of phase-known data (results not shown).

### Generation of missing data

Data sets containing unknown alleles were generated from the original data via the following procedure:

1. Each individual is selected in turn.

2. For each locus a random number between 0 and 100 is generated.

3. If this random number falls below the desired percentage of unknowns, both of the individual's alleles at the locus in question are redefined as unknown. This ensures that all unknowns appear in homologous pairs.

4. The process is repeated until all loci for all individuals are exhausted.

Thus the desired percentage of unknown alleles is achieved globally, and the percentage of missing data at each locus may vary. Three additional sets of data were generated from each of the two original sets in this way, with 10%, 20% and 30% missing data respectively, giving eight data sets in all for the principal component of the study.

### Expectation-Maximisation algorithm

For known gametic phase, HFE is a straightforward process of counting the constituent haplotypes in the sample. For the case where the gametic phase is unknown, maximum-likelihood haplotype frequencies are computed using the EM algorithm. The particular implementation used here for the finding of the haplotype frequencies is similar to that outlined by Excoffier and Slatkin [6]. The operation of the algorithm is based on the assumption of HWE, though as mentioned above, the method has been found to be quite robust in the presence of deviations from HWE [13].

#### Implementation of the EM algorithm

Missing data in a sample necessitate alterations to the implementation for complete data of the EM-based algorithm. When all alleles in an individual are known, there exist *c*_*j *_possible genotypes consistent with this phenotype where


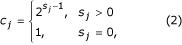


and *s*_*j *_is the number of heterozygous loci in phenotype *j*. However, when unknown alleles appear at a locus, the situation is considerably more complex. In this case each unknown allele may take on the identity of any of the alleles observed at that locus. We require that unknown alleles always appear in pairs – the amplification of one allele only would result in the appearance of a homozygote which may bias results. Thus if there are *N*_*i *_distinct alleles (forms) observed at locus *i *in the entire sample, the number of possible *complete *phenotypes consistent with the observed phenotype is increased by a factor of *N*_*i*_(*N*_*i *_+ 1)/2 by the presence of an unknown site. This factor is the number of ways of selecting two alleles from a pool of *N*_*i *_distinct alleles when repetition is allowed. Thus the number of possible complete phenotypes given by phenotype *j *is given by


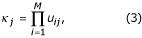


where *M *is the number of loci in the sample and


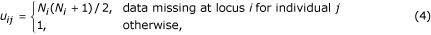


where *N*_*i *_is the number of distinct alleles observed in the sample at locus *i*. For each possible complete phenotype *i *of the *κ*_*j *_complete phenotypes possible for individual *j*, there exist *c*_*i *_possible genotypes, as given by Equation 2. Thus the number possible complete *genotypes *for phenotype *j *is given by


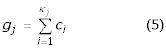


Then, following [6], the probability *P*_*j *_of the *j*^*th *^phenotype, assuming random mating, is given by:


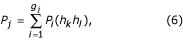


where *P*_*i*_(*h*_*k*_*h*_*l*_)is the probability of the *i*^*th *^genotype made up of haplotypes *k *and *l*, and


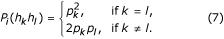


where *p*_*k *_and *p*_*l *_are the population frequencies of the *k*^*th *^and *l*^*th *^haplotypes.

#### Expectation step

At the *t*^*th *^step of the EM iterative process, the probability of resolving each phenotype into the different possible genotypes is given by:


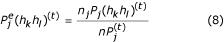


where *n*_*j *_is the number of individuals with phenotype *j*, and *n *is the total number of individuals in the sample. Thus *n*_*j*_/*n *is the proportion of the total sample that has phenotype *j*, and *P*_*j*_(*h*_*k*_*h*_*l*_)/*P*_*j *_is the conditional probability of the particular genotype given the phenotype.

#### Maximisation step

The haplotype frequencies are then computed using a form of gene-counting [16,17] :


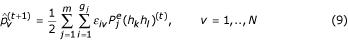


where *N *is the number of globally distinct haplotypes (the number of different possible haplotypes in the sample), 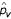 is the frequency of haplotype *v, m *is the number of distinct phenotypes in the sample, and *ε*_*iv *_is equal to the number of times haplotype *v *appears in genotype *i*.

### Generation of confidence intervals

The technique of bootstrapping [18] was used to generate CIs about the point haplotype frequencies estimated from the phase-unknown data. Specifically, the percentile bootstrap approach was used.

## Authors' contributions

EDK carried out the main programming work, performed the tests and drafted the manuscript. FS designed the population generation tool and assisted in the programming effort. RM assisted in the drafting of the manuscript and provided the SNP data. All authors read and approved the final manuscript.

## Figures and Tables

**Table 1:** *D *(*h*, 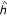) for 7 loci SNP data. Measure of discrepancy between phase-known and phase-unknown frequency predictions for seven loci biallelic data, 536 individuals.

% missing alleles	*D *(*h*, 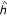 )	% increase from complete data
0	0.043822	0
10	0.059222	35
20	0.069017	57
30	0.075998	73

**Table 2:** RCI for 7 loci SNP data. Ratio of extent of 95% CI for missing data sets compared to complete data set for seven loci biallelic case, 536 individuals.

% missing alleles	RCI
0	1.0
10	1.102024
20	1.243451
30	1.469974

**Table 3:** *D *(*h*, 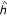) for multiallelic data. Measure of discrepancy between phase-known and phase-unknown frequency predictions for multiallelic data.

% missing alleles	*D *(*h*, 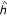 )	% increase from complete data
0	0.106954	0
10	0.151494	42
20	0.170971	60
30	0.213888	100

**Table 4:** RCI for multiallelic data. Ratio of extent of 95% CI for missing data sets compared to complete data set for multiallelic case.

% missing alleles	RCI
0	1.0
10	1.162630
20	1.315285
30	1.470215

**Table 5:** *D *(*h*, 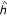) for smaller samples. Measure of discrepancy between phase-known and phase-unknown frequency predictions for smaller samples randomly selected from larger data sets.

Data set	# individuals	% missing alleles	*D *(*h*, 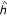 )	% increase from complete data
7 loci SNP	300	0	0.049354	0
7 loci SNP	300	10	0.067410	37
7 loci SNP	100	0	0.104105	0
7 loci SNP	100	10	0.147034	41
7 loci SNP	50	0	0.155912	0
7 loci SNP	50	10	0.229097	47
multiallelic	300	0	0.153865	0
multiallelic	300	10	0.202678	32
multiallelic	100	0	0.227170	0
multiallelic	100	10	0.238658	5
multiallelic	50	0	0.320917	0
multiallelic	50	10	0.372827	16

**Table 6:** *D *(*h*, 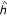) for low LD data. Measure of discrepancy between phase-known and phase-unknown frequency predictions for low LD data.

% missing alleles	*D *(*h*, 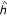 )	% increase from complete data
0	0.090178	0
10	0.144290	60

## References

[B1] Michalatos-Beloin S, Tishkoff SA, Bentley KL, Kidd KK, Ruano G (1996). Molecular haplotyping of genetic markers 10 kb apart by allele-specific long-range PCR. Nucleic Acids Res.

[B2] Hill WG (1974). Estimation of linkage disequilibrium in randomly mating populations. Heredity.

[B3] Dempster AP, Laird NM, Rubin DB (1977). Maximum likelihood from incomplete data via the EM algorithm. J Royal Stat Soc B.

[B4] Long JC, Williams RC, Urbanek M (1995). An E-M algorithm and testing strategy for multiple-locus haplotypes. Am J Hum Genet.

[B5] Hawley ME, Kidd KK (1995). HAPLO: A program using the EM algorithm to estimate the frequencies of multi-site haplotypes. J Hered.

[B6] Excoffier L, Slatkin M (1995). Maximum-likelihood estimation of molecular haplotype frequencies in a diploid population. Mol Biol Evol.

[B7] Fallin D, Schork NJ (2000). Accuracy of haplotype frequency estimation for biallelic loci, via the Expectation-Maximisation algorithm for unphased diploid genotype data. Am J Hum Genet.

[B8] Tishkoff SA, Pakstis AJ, Ruano G, Kidd KK (2000). The accuracy of statistical methods for estimation of haplotype frequencies: An example from the CD4 locus. Am J Hum Genet.

[B9] Zhao JH, Lissarrague S, Essioux L, Sham PC (2002). GENECOUNTING: haplotype analysis with missing genotypes. Bioinformatics.

[B10] SNPHAP A program for estimating frequencies of large haplotypes of SNPs. http://www-gene.cimr.cam.ac.uk/clayton/software/snphap.txt.

[B11] Kirk KM, Cardon LR (2002). The impact of genotyping error on haplotype reconstruction and frequency estimation. Eur J Hum Genet.

[B12] Lewontin RC (1964). The interaction of selection and linkage I. General considerations; heterotic models. Genetics.

[B13] Niu T, Qin ZS, Xu X, Liu JS (2002). Bayesian haplotype inference for multiple linked single-nucleotide polymorphisms. Am J Hum Genet.

[B14] Kerem B, Rommens JM, Buchanan JA, Markiewicz D, Cox TK, Chakravarti A, Buchwald M, Tsui LC (1989). Identification of the cystic fibrosis gene: genetic analysis. Science.

[B15] Guo SW, Thompson EA (1992). Performing the exact test of Hardy-Weinberg proportion for multiple alleles. Biometrics.

[B16] Ceppellini R, Siniscalco M, Smith CAB (1955). The estimation of gene frequencies in a random mating population. Ann Hum Genet.

[B17] Smith CAB (1957). Counting methods in genetical statistics. Ann Hum Genet.

[B18] Efron B, Tibshirani RJ (1993). An Introduction to the Bootstrap.

